# Proptosis Revealing a Neurological Form of IgG4-Related Disease: A Case Report and Literature Review

**DOI:** 10.7759/cureus.113742

**Published:** 2026-07-31

**Authors:** Karim Baayoud, Zakariae Benyaich, Achref Miry, Kadiri A Naji, Mohamed Lmejjati, Sanae Abbaoui

**Affiliations:** 1 Neurosurgery, Mohammed VI University Hospital of Agadir, Agadir, MAR; 2 Pathology, Mohammed VI University Hospital of Agadir, Agadir, MAR

**Keywords:** cavernous sinus lesions, igg4-related disease, orbital invasion tumor, orbital proptosis, tentorium lesion

## Abstract

IgG4-related disease (IgG4-RD) is a chronic, immune-mediated fibro-inflammatory condition characterized by tissue infiltration of IgG4-positive plasma cells, with a continually expanding spectrum of clinical manifestations. Neurological involvement is uncommon, and imaging findings are often nonspecific, frequently delaying diagnosis. The condition generally carries a favorable prognosis due to its responsiveness to corticosteroid therapy.

We report the case of a 30-year-old man presenting with a nine-month history of progressive, painless left-sided proptosis and eyelid swelling, without focal neurological deficits. Imaging revealed an orbital lesion extending posteriorly into the intracranial compartment. Orbital decompression via an eyebrow incision was performed, and histopathological examination confirmed IgG4-RD. The patient was subsequently treated with systemic corticosteroids, with a favorable clinical outcome.

This case highlights the diagnostic challenge posed by IgG4-related orbital disease (IgG4-ROD) with neurological involvement, given the limited specificity of imaging in distinguishing it from other orbital and skull-base pathologies. It underscores the importance of maintaining a broad differential diagnosis for proptosis and the essential role of histopathological confirmation in guiding timely, appropriate management.

## Introduction

IgG4-related disease (IgG4-RD) is a rare immune-mediated condition characterized by sclerosing inflammation and fibrosis, resulting from infiltration by IgG4-positive plasma cells [1]. The pancreas is the most commonly described location, though other organs can also be involved, as extensively documented [1,2].

Neurological involvement is rare and has been reported in cases with hypophysitis, pachymeningitis, pseudotumors, perineural spread, and brain parenchymal infiltration [3,4]. Diagnosing intracranial IgG4-RD is often delayed because its imaging features are nonspecific and resemble those of more common infiltrative and inflammatory diseases, such as meningioma, lymphoma, metastatic lesions, inflammatory pseudotumor, and granulomatous disease [5].

Recognizing IgG4 disease as a potential cause of hypertrophic pachymeningitis can avoid significant complications, including unnecessary surgery. IgG4-RD typically responds well to corticosteroids, and fibrotic changes can improve with treatment [6].

This case report highlights the importance of early recognition of the orbital and neurological form of IgG4-RD and individualizing the differential diagnosis that should be considered in the management of this rare inflammatory condition. 

This case is reported to highlight the rare presentation of IgG4-RD as an isolated orbital mass with posterior extension into the intracranial compartment, a neurological variant that remains underrecognized and is easily mistaken for more common orbital or skull-base pathologies on imaging alone. Emphasizing histopathological confirmation in such cases may help avoid diagnostic delay and unnecessary invasive procedures.

## Case presentation

A 30-year-old male patient with no medical history, who reported a nine-month history of painless progressive left proptosis, unaccompanied by visual loss. Associated symptoms included mild left-sided headaches (Visual Analog Scale was 3/10), with no nausea, vomiting, limb weakness, seizures, fever, or deterioration of general condition. Two weeks before admission, a notable worsening of both the proptosis and headaches prompted the patient to seek medical attention.

On clinical examination, the patient was conscious and oriented, with no focal motor or sensory deficits. Ophthalmological assessment revealed painless, non-pulsatile left axial proptosis with associated periorbital edema. Ocular motility and visual acuity were intact bilaterally, and fundoscopic examination showed no evidence of papilledema.

**Figure 1:**
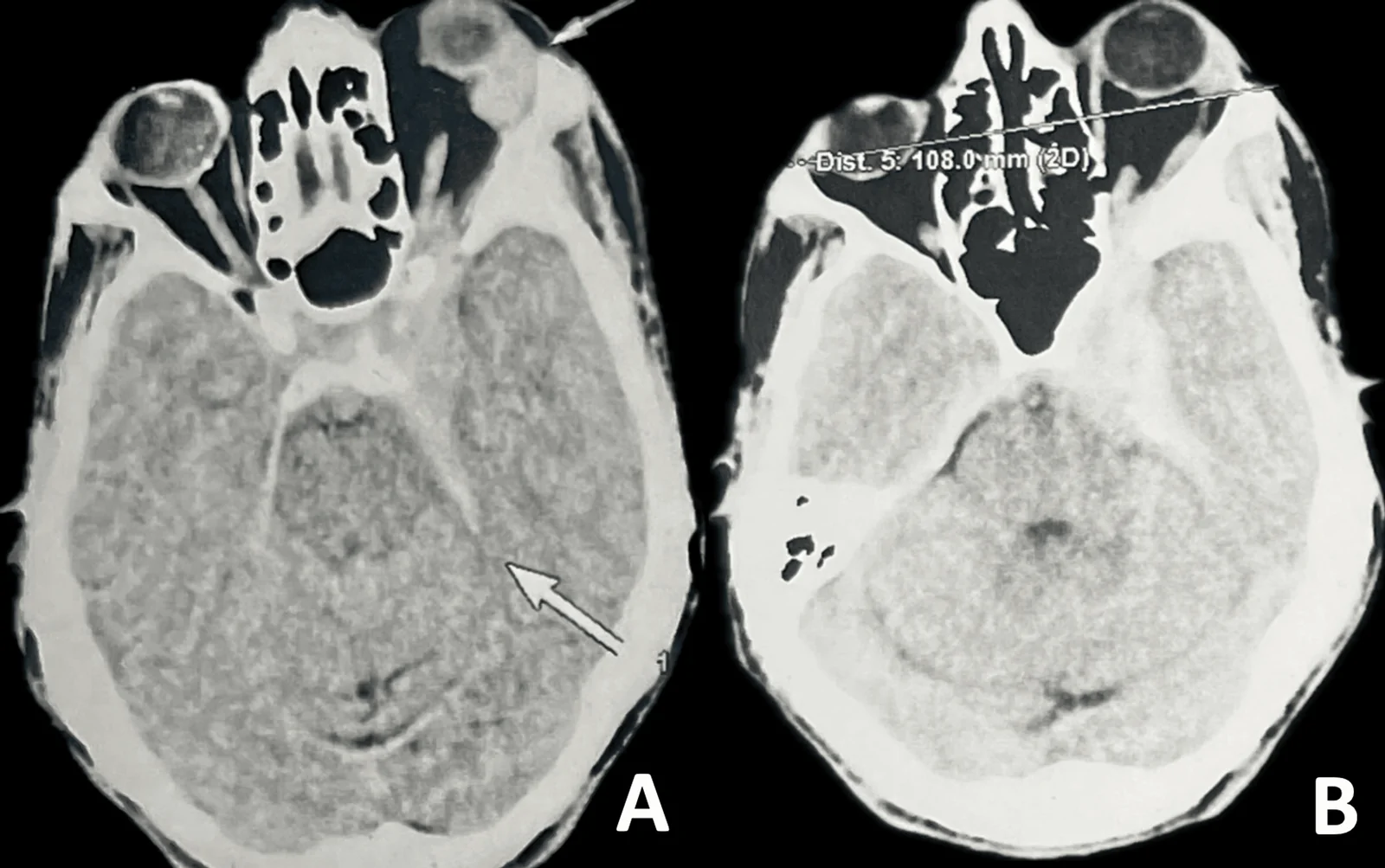
Brain CT scan with and without contrast enhancement (axial views) (A) Pre-contrast axial CT: A well-defined, rounded, isodense lesion is identified within the left orbit, exerting mass effect on adjacent orbital structures (thin arrow). A second isodense lesion with irregular margins is noted extending posteriorly from the orbital apex to the lateral wall of the cavernous sinus, with involvement of the left tentorial leaflet (thick arrow). (B) Post-contrast axial CT: Both lesions demonstrate avid and homogeneous contrast enhancement, consistent with highly vascularized or inflammatory pathology. Measurement along the external bicanthal line confirms grade III proptosis.

Computed tomography (CT) of the brain and orbits demonstrated a well-defined, rounded, isodense lesion occupying the left orbital lateral wall, exerting significant mass effect on adjacent structures and accounting for grade III proptosis. A second isodense lesion with irregular margins was identified, extending posteriorly from the orbital apex to the lateral wall of the cavernous sinus, with an attachment to the left tentorial leaflet. Both lesions demonstrated avid enhancement following contrast administration (Figure 1).

**Figure 2:**
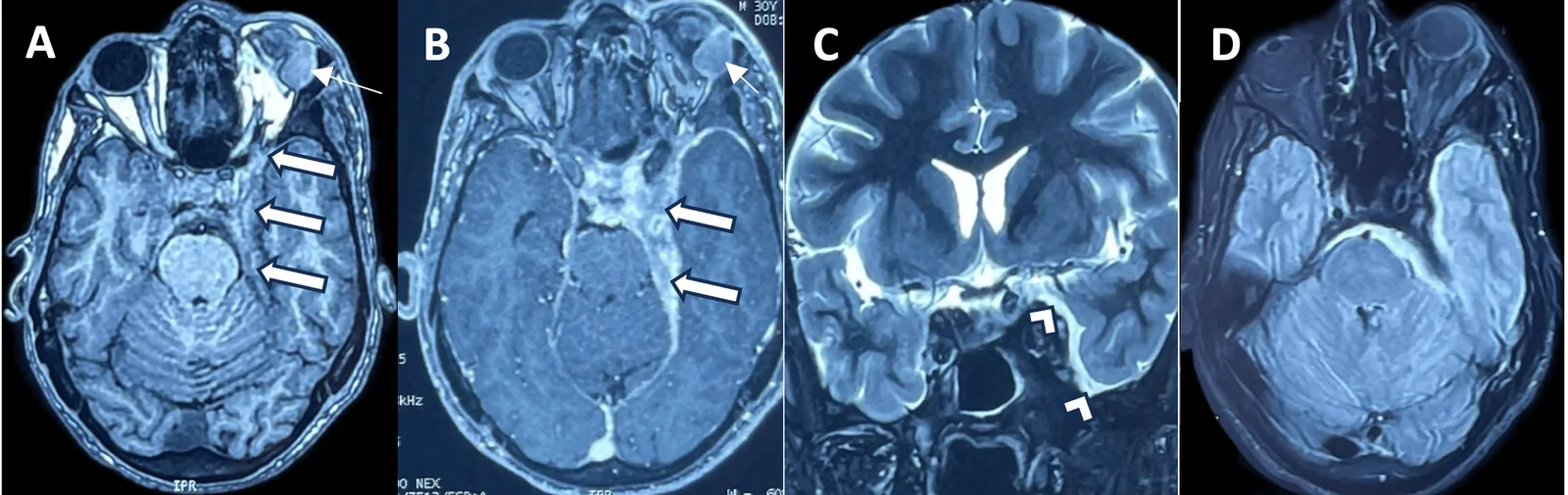
Brain MRI imaging demonstrating orbital and intracranial lesions. (A) Axial T1 pre-contrast: Two isointense lesions are identified: a well-defined round lesion occupying the left orbit (thin arrow), and a second lesion extending posteriorly from the orbital apex to the lateral wall of the cavernous sinus, with involvement of the left tentorium leaflet and an associated dural tail sign (thick arrow). (B) Axial T1 post-contrast: The orbital lesion (thin arrow) doesn't enhance after gadolinium injection. The intracranial lesion demonstrates avid and homogeneous enhancement, with conspicuous enhancement of the dural tail, suggestive of dural-based pathology (thick arrow). (C) Coronal T2: Diffuse thickening of the dura overlying the temporal fossa is identified, extending medially to involve the cavernous sinus (arrowhead). (D) Fluid-attenuated inversion recovery (FLAIR): A hyperintense lesion is demonstrated extending from the middle cranial fossa through the tentorial hiatus to the cerebellopontine angle cistern.

Subsequent magnetic resonance imaging (MRI) of the brain confirmed two lesions appearing isointense on T1-weighted sequences and hyperintense on T2-weighted sequences, with vivid post-contrast enhancement. The intracranial component appeared to extend from the middle cranial fossa to the posterior fossa through the tentorial hiatus (Pacchioni's foramen) (Figure 2). Laboratory investigations revealed isolated lymphopenia (980 cells/mm³), with no other significant abnormalities.

**Figure 3:**
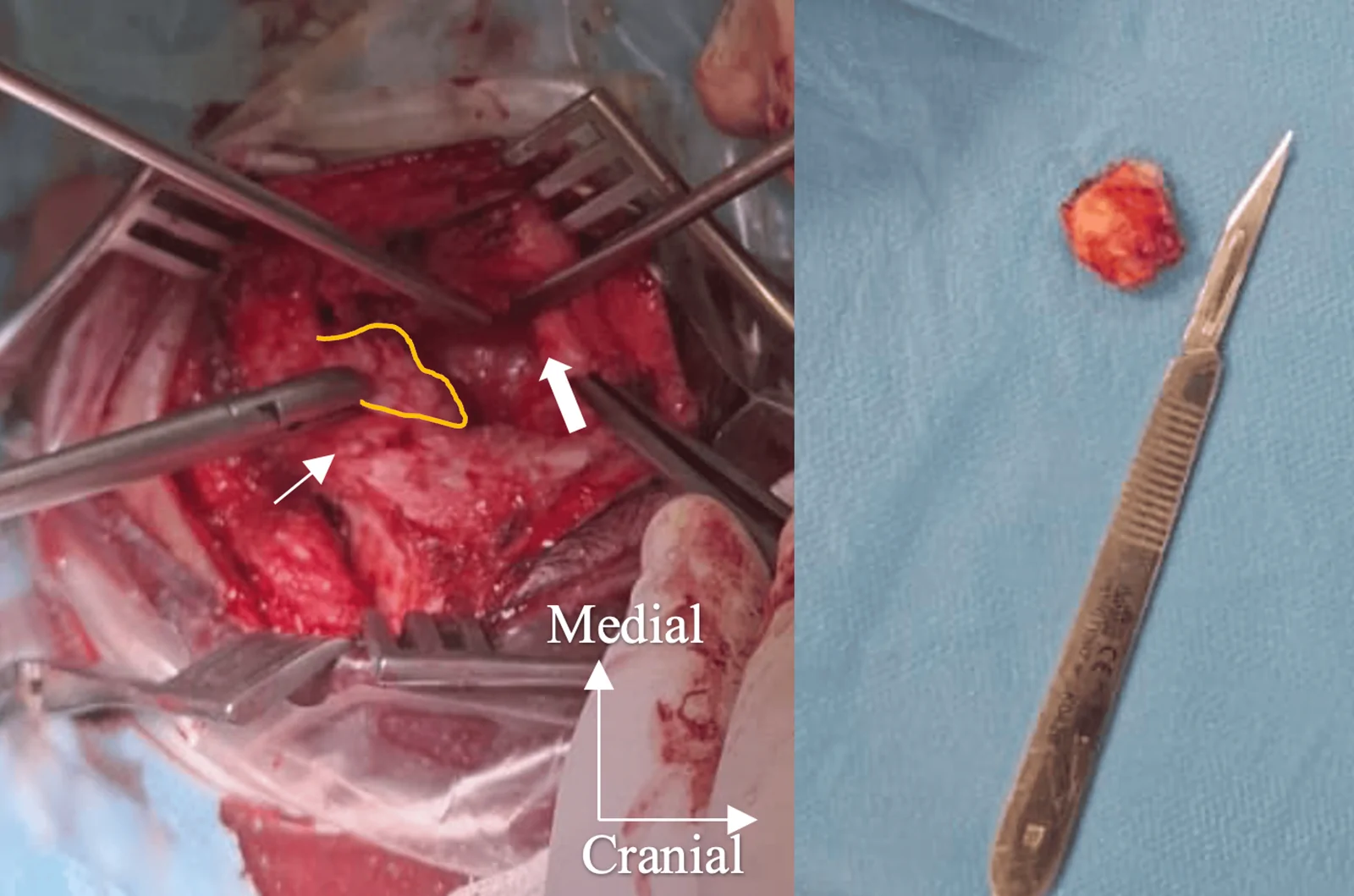
Intraoperative image of the transorbital approach via an eyebrow incision showing the tumor (highlighted in yellow), the periorbital tissue (thick arrow), and the frontal process of the zygomatic bone (thin arrow).

The patient underwent surgical excision of the orbital lesion via an eyebrow incision approach (Figure 3), combined with orbital decompression. Intraoperatively, the lesion appeared pearly white and was adherent to the periorbital tissue, with no attachment to the orbit; it was slightly firm in consistency and non-hemorrhagic. We proceeded to gradually dissect it using a dissector before excising it. The procedure was uneventful, and the postoperative course was uncomplicated.

**Figure 4:**
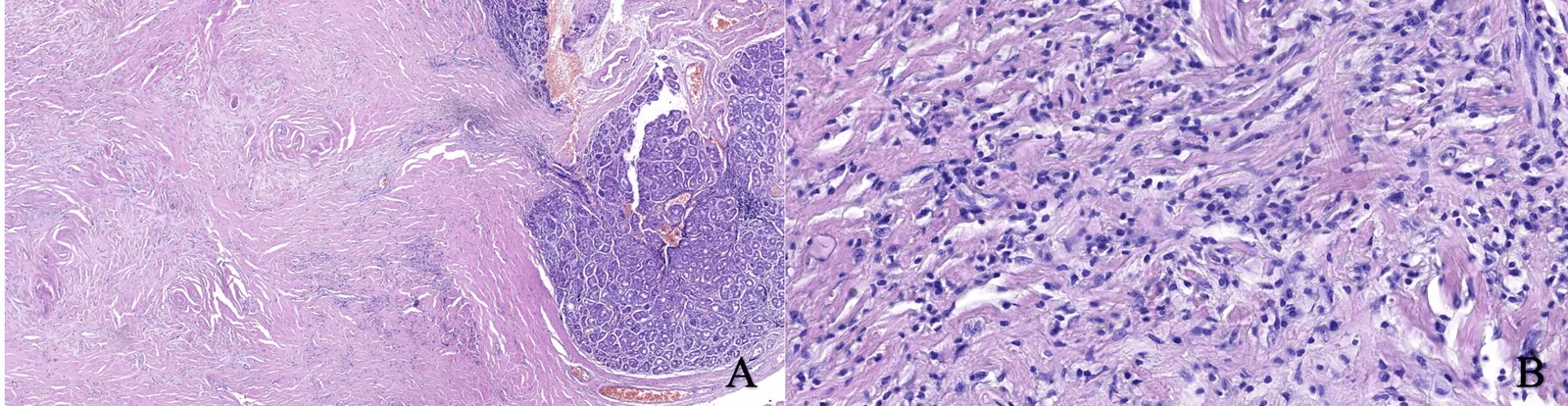
Histopathological and immunohistochemical features of the IgG4 disease (A) Microscopic photograph showing a sclerotic process focally encasing the lacrimal glands (HE; 200X). (B) Microscopic photograph showing the presence of an infiltrate containing numerous plasma cells (HE; 400X).

Histopathological examination of the resected specimen confirmed the diagnosis of IgG4-RD (Figure 4).

**Figure 5:**
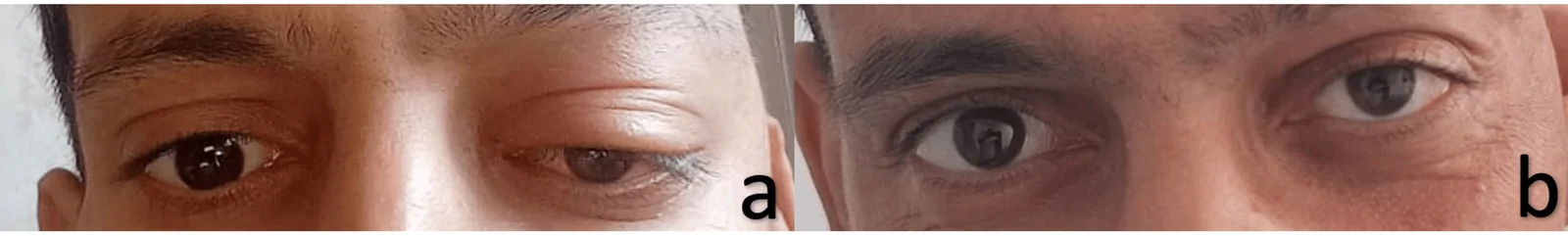
Comparative images showing the regression of left proptosis at four months. (a) Proptosis before surgery; (b) Regression of proptosis four months after surgery.

The patient was subsequently transferred to the internal medicine department to initiate systemic corticosteroid therapy. At four months postoperatively, a significant regression of the proptosis was observed (Figure 5).

## Discussion

IgG4-RD is a chronic, immune-mediated fibro-inflammatory condition characterized by sclerosing inflammation resulting from the pathological infiltration of IgG4-positive plasma cells across one or multiple organ systems. Although only recently recognized as a distinct clinicopathological entity, its epidemiology is becoming better defined; a large US population-based study estimated a prevalence of 5.3 per 100,000 individuals and an annual incidence of 1.4 per 100,000 person-years [7].

The condition predominantly affects middle-aged adults, with peak onset occurring between the fifth and sixth decades of life [8]. Pediatric cases remain exceptional [9]. The sex distribution is organ-dependent; in head and neck involvement, a female predominance has been consistently reported [10]. Virtually any organ may be affected, with the pancreas being the most frequently involved site. Other reported locations encompass the salivary glands, lymph nodes, and both the peripheral and central nervous systems, either concurrently or in a sequential fashion [11].

Neurological manifestations are among the rarest expressions of IgG4-RD, as confirmed by four major epidemiological cohort studies [12-14]. When neurological involvement does occur, the most characteristic manifestations include orbital disease (IgG4-related orbital disease (IgG4-ROD)), hypertrophic pachymeningitis, and hypophysitis [15].

Regarding pathogenesis, genetic susceptibility has been linked to specific loci, including HLA-DRB1 and FC-γ receptor IIb regions, while cigarette smoking represents the sole identified modifiable risk factor [16,17].

From a histopathological standpoint, the diagnosis rests on three cardinal tissue findings: a dense lymphoplasmacytic infiltrate, storiform fibrosis, and obliterative phlebitis [18]. At the cellular level, IgG4-RD is driven by aberrant immune activation involving B cells, plasmablasts, and multiple T-cell subsets [6,8]. B cells occupy a central pathogenic role, as demonstrated both by the disease's responsiveness to B-cell depletion therapy and by the identification of self-antigens capable of sustaining clonal B-cell expansion [19]. Plasmablasts, present in high concentrations in affected tissues and peripheral blood, serve as a reliable marker of disease activity [20]. The detection of oligoclonal IgG4 bands in cerebrospinal fluid obtained from patients with IgG4-related hypertrophic pachymeningitis further supports the hypothesis of a localized, antigen-driven immune response [21].

The clinical presentation varies according to the anatomical site of involvement. IgG4-ROD typically manifests with progressive eyelid swelling and proptosis and may additionally present with diplopia, restricted ocular motility, and reduced visual acuity [6]. Hypertrophic pachymeningitis results from dural thickening and may cause headache, cranial nerve palsy, and visual disturbances, including diplopia and visual impairment [6]. Hypophysitis most commonly presents as hypopituitarism, and isolated cases of diabetes insipidus have been documented [6].

From a radiological perspective, IgG4-RD can present in various ways depending on the clinical presentation. In pachymeningitis, lesions may appear as either linear dural thickening or focal dural masses and may be localized or diffuse. On MRI, they are typically isointense on T1-weighted sequences and hypointense on T2-weighted sequences, the latter reflecting the underlying fibrosis. Post-gadolinium enhancement is variable, presenting as patchy or uniform. Associated findings may include cranial nerve sheath thickening or osseous remodeling without frank bone destruction [22]. IgG4-RD hypophysitis is uncommon, but in the literature, the most commonly reported imaging features include nonspecific enlargement of the pituitary gland, with or without infundibulum enlargement, visible on MRI. It mostly enhances after a gadolinium injection [23-25]. IgG4-ROD appears as a tumefactive lesion with abnormal, often enhancing signals, reminiscent of an “orbital pseudotumor” [26].

Diagnostic criteria were formally updated in 2020 by the Japanese Society, which introduced the revised comprehensive diagnostic criteria encompassing three domains: (1) clinical and radiological features, defined by the presence of organ swelling, masses, or nodules characteristic of IgG4-RD, with isolated lymph node swelling excluded in single-organ presentations; (2) serological criteria, defined by serum IgG4 concentrations exceeding 135 mg/dL; and (3) pathological criteria, requiring at least two of the following: dense lymphoplasmacytic infiltration with associated fibrosis, an IgG4-positive to IgG-positive plasma cell ratio exceeding 40%, or the presence of characteristic tissue fibrosis in a storiform pattern or obliterative phlebitis [27].

Treatment is primarily based on corticosteroid therapy, conventionally initiated at 0.5 to 0.6 mg/kg/day and progressively tapered over a period of three to six months. Responsiveness to glucocorticoids is itself considered a hallmark feature of IgG4-RD, and fibrotic tissue changes typically begin to regress within the first weeks of treatment, accompanied by a corresponding decline in serum IgG4 levels [28-31]. Corticosteroid therapy achieves a favorable clinical response in approximately 97% of cases; however, the risk of relapse following dose reduction or discontinuation remains substantial, reported at approximately 33%, underscoring the necessity for prolonged clinical and biological surveillance [30-32].

## Conclusions

IgG4-RD is a chronic, multisystem, immune-mediated fibro-inflammatory disorder characterized by sclerosing inflammation and progressive fibrosis resulting from pathological tissue infiltration by IgG4-positive plasma cells. Although the disease can involve virtually any organ system, neurological manifestations are rare; the most frequently reported are hypertrophic pachymeningitis and hypophysitis. Several medical societies have established diagnostic criteria based on clinical, radiological, histopathological, and serological findings. Corticosteroid therapy remains the cornerstone of treatment and typically produces a rapid and substantial clinical response; however, relapses are frequently observed upon dose tapering or discontinuation, necessitating careful long-term follow-up.
